# Biomarkers of autoimmunity and beta cell metabolism in type 1 diabetes

**DOI:** 10.3389/fimmu.2022.1028130

**Published:** 2022-10-27

**Authors:** Mei-Ling Yang, Richard G. Kibbey, Mark J. Mamula

**Affiliations:** ^1^ Section of Rheumatology, Allergy and Immunology, Department of Internal Medicine, Yale University, New Haven, CT, United States; ^2^ Section of Endocrinology, Department of Internal Medicine, Yale University, New Haven, CT, United States

**Keywords:** type 1 diabetes, glucose metabolism, posttranslational modifications, biomarkers, neoepitopes

## Abstract

Posttranslational protein modifications (PTMs) are an inherent response to physiological changes causing altered protein structure and potentially modulating important biological functions of the modified protein. Besides cellular metabolic pathways that may be dictated by PTMs, the subtle change of proteins also may provoke immune attack in numerous autoimmune diseases. Type 1 diabetes (T1D) is a chronic autoimmune disease destroying insulin-producing beta cells within the pancreatic islets, a result of tissue inflammation to specific autoantigens. This review summarizes how PTMs arise and the potential pathological consequence of PTMs, with particular focus on specific autoimmunity to pancreatic beta cells and cellular metabolic dysfunction in T1D. Moreover, we review PTM-associated biomarkers in the prediction, diagnosis and in monitoring disease activity in T1D. Finally, we will discuss potential preventive and therapeutic approaches of targeting PTMs in repairing or restoring normal metabolic pathways in pancreatic islets.

## Introduction – overview of beta cell metabolism associated with inflammatory PTMs

Posttranslational modifications (PTMs) change the properties of a protein and shape its biological functions (1). Various pathways altered by PTMs that arise from tissue inflammation have been closely linked to numerous disorders including cancer and autoimmune diseases (2, 3). Type 1 diabetes (T1D) is a chronic autoimmune disease characterized by altered glucose sensing and insulin response resulting that may arise from the immune attack of insulin-secreting beta cells in the pancreas. In addition, tissue specific properties of pancreatic islets, and beta cells in particular, may contribute to the autoimmune pathology (4, 5). However, we are reminded that T1D, similar to other autoimmune syndromes, are multifactorial in origin, including a role for genetics, stochastic factors, and environmental influences in the onset and progression of disease.

It is clear that some, or many, tissue specific PTMs may not be expressed in the thymus in the course of immune tolerance induction, though clear studies of specific PTMs are lacking in fully understanding central tolerance to modified proteins. However, T cells specific to PTM determinants can escape selection from the immune system, providing the potential for the modified proteins to be recognized as neo-antigens and contribute to autoimmunity. As one clear example, the role of citrullination PTM has been extensively studied in rheumatoid arthritis (RA). As with T1D, RA is also a chronic autoimmune disease characterized by inflammation of the target tissue, connective tissue in the joints, and more than 100 citrullinated proteins have been identified from RA synovium (6). Anti-citrullinated protein antibodies (ACPAs), present before the early onset of RA and correlate with disease severity, are routinely used for the diagnosis of RA for over a decade (7, 8). Similarly, citrullination has recently become a relevant PTM in T1D pathology (9). Accumulating evidence has identified significant numbers of citrullinated islet proteins, including proteins in the glucose and insulin metabolic pathways. These citrulline PTM proteins elicit vigorous B and T cell autoimmune responses in both human T1D and NOD murine disease, including glutamic acid decarboxylase 65 (GAD 65), 78-kDa glucose-regulated protein (GRP78) (also called BiP, HSP5a), islet antigen-2 (IA2), islet-specific glucose 6 phosphatase catalytic subunit-related protein (IGRP), islet amyloid polypeptide (IAPP) and glucokinase (10). Indeed, several other inflammatory PTMs also play the vital roles in the progression of T1D, including deamidation, oxidation and carbonylation (11). Not surprisingly, several enzymes responsible for forming, repairing and/or regulating PTMs such as peptidylarginine deiminase (PADs), antioxidant enzymes, catalase, glutathione peroxidase 1 (GPx1), and superoxide dismutase (SOD) are also found to modulate T1D autoimmunity and glucose and insulin metabolism. However, it is important to realize that it is not clearly known if specific PTMs are a cause of pathology or a consequence of pathology in human T1D. Animal models and ex-vivo studies have been partly useful in defining the roles of PTMs in disease but do not perfectly reflect human disease.

Beta cells have very specific intracellular pathways that are involved in coupling the metabolism of glucose to the release of insulin. Many components of these pathways are subject to modification by PTMs, including citrullination. The details of how beta cell metabolism is coupled to insulin secretion through oscillatory activation of the phosphoenolpyruvate (PEP) cycle to close K_ATP_ channels have been recently reviewed in depth (12) and will only be summarized here.

Glucose enters the rodent beta cell through glucose transporter 2 (Glut2) and is introduced into glycolytic metabolism relative to its concentration in the plasma by the activity of glucokinase (GK). GK is not product inhibited unlike the other hexokinases and has an EC_50_ for glucose in the physiologic range. The glucose carbons then flow through glycolysis and enter the mitochondria as pyruvate and follows one of two pathway fates. The first pathway is the more familiar pyruvate dehydrogenase pathway (where pyruvate is converted to acetyl CoA), which is ultimately oxidized to CO_2_ by the TCA cycle and electron transport chain in the process of oxidative phosphorylation (OXPHOS). OXPHOS supports the basal ATP requirements of the cell. As the ATP/ADP ratio reaches its thermodynamic equilibrium the OXPHOS progressively slows in the process known as ADP privation where the mitochondrial membrane potential hyperpolarizes, and TCA cycle intermediate accumulate (13). In particular, acetyl CoA increases in response to the high matrix NADH/NAD^+^ and mitochondrial GTP production is increased driven by the high ATP/ADP ratio (via antiparallel collaboration of the ATP and GTP isoforms of succinyl CoA synthesis) (14). Increased acetyl CoA activates pyruvate carboxylase diverting pyruvate into anaplerotic synthesis of oxaloacetate (OAA) consuming an ATP in the process. In the mitochondrial matrix, OAA is converted into phosphoenolpyruvate (PEP) coupled to GTP hydrolysis by the mitochondrial isoform of PEPCK (PCK2) (15). Thus, the newly made, highly energetic PEP exits the matrix where it is used to raise the ATP/ADP ratio *via* hydrolysis back to pyruvate by pyruvate kinase (PK). The cycle from pyruvate to OAA to PEP and back to pyruvate is known as the PEP cycle. Because PK raises the ATP/ADP ratio more than the mitochondria bioenergetics permit and because it is physically localized to the K_ATP_ channel, it closes K_ATP_ channels to depolarize the plasma membrane to allow Ca^2+^ to enter to stimulate insulin release (13).

The cellular workload from membrane depolarization and insulin release increases ATP hydrolysis resetting the system and returning control over ATP synthesis back to OXPHOS. Subsequent cycles of such coordinated metabolic and electrical oscillations are partially entrained by the changes in cellular work as well as the generation of fructose-1, 6-bisphosphate (F16BP) by the PFK1/PFKFB3 system. F16BP allosterically activates PK and supports mitochondrial ADP privation and K_ATP_ closure. Many of the components of this glucose-sensing mechanism in beta cell are modified by PTMs triggered by beta cell stress (**Table 1**).

**Table 1:** Potential immunometabolic biomarkers in type 1 diabetes.

Target proteins	Involved pathways	Relevant PTMs and pathological roles	References
GLUT1	glucose intake	reduces diabetogenic CD8+ T cells by GLUT1 blockade	(16)
SGLT2	glucose intake	maintains blood glucose level by SGLT2 inhibitors	(17)
GK	glycolysis	alters GK activity by ubiquitination, SUMOylation and citrullination diminishes insulin secretion by citrullination increased citrullinated GK in islets before the onset of hyperglycemia autoantibodies against GK and citrullinated GK in NOD and T1D serum autoreactive T cells against citrullinated GK peptide in T1D patients	(10, 18, 19) (10) (10) (10) (10)
PFKFB3	glycolysis	inhibits diabetogenic CD4+ T cells response to beta cell antigens by PFKFB3 inhibitor, PFK15 delays diabetic onset in adoptive transfer model of T1D by PFKFB3 inhibitor, PFK15 increased PFKFB3 expression in T1D beta cells	(20) (20) (21)
Enolase	glycolysis	loss expression of neuron-specific enolase in T1D decreased α-enolase expression in T1D renal glomeruli	(22) (23)
PK	glycolysis	alters PK activity by phosphorylation, acetylation, citrullination, methylation, succinylation and glycosylation decreased PKM1 and PKM2 expression in T1D renal glomeruli protects diabetes by PKM2 activation	(24–26) (23) (27)
PDH	TCA cycle	alters PDH activity by phosphorylation, acetylation and succinylation beta cell-specific PDH knockout mouse develops increased blood glucose level and decreases GSIS	(28, 29) (30)
PC	TCA cycle	overexpression of PC in INS-1 cells increases insulin secretion and cell proliferation protects human beta cells from inflammation and nitrosative stress	(31) (32, 33)
PDIA1/P4Hb	protein folding	ablation of the thioredoxin activity prevents the refolding of denatured and reduced proinsulin increased carbonylated PDIA1/P4Hb in islets before the onset of hyperglycemia autoantibodies against PDIA1/P4Hb and carbonylated PDIA1/P4Hb in NOD and T1D serum autoreactive T cells against carbonylated PDIA1/P4Hb peptide in T1D patients increased PDIA1/P4Hb level in NOD and T1D plasma	(34) (35) (35) (35) (36)
GRP78	protein folding	autoantibodies against GRP78 and citrullinated GRP78 in NOD and T1D serum autoreactive T cells against citrullinated GRP78 peptide in T1D patients	(37, 38) (37)
Insulin	glucose homeostasis	PTMs, citrullination, deamidation, chlorination and oxidation, increase HLA-A*02:01- binding affinity to insulin-B-derived epitopes autoantibodies against oxidized insulin in T1D serum autoreactive T cells against deamidated insulin B30-C13 in T1D patients	(39–41) (42–44) (45)
GAD65	glutamate metabolism	autoantibodies against citrullinated and deamidated GAD65 peptides in T1D patients autoreactive T cells against citrullinated and deamidated GAD65 peptides in T1D patients	(46–49) (49–52)
IGRP	glucose metabolism	autoreactive T cells against citrullinated IGRP peptides in T1D patients	(50)
IAPP	glycemic regulator	autoreactive T cells against IAPP and citrullinated IAPP peptides in NOD and T1D serum	(51–53)
IA2	insulin-signaling pathway regulator	autoantibodies against IA2 and deamidated IA2 in T1D serum autoreactive T cells against IA2 and deamidated IA2 peptides in T1D patients	(54) (50, 55)

GLUT1, glucose transporter 1; SGLT2, sodium-glucose co-transporter-2; GK, glucokinase; PFKFB3, 6-phosphofructo-2-kinase/fructose-2,6- biphosphatase 3; PK, pyruvate kinase; PDH, Pyruvate dehydrogenase; PC, Pyruvate carboxylase; PDIA1/P4Hb, Protein disulfide isomerase A1 (PDIA1)/prolyl-4-hydroxylase beta; GRP78, glucose-regulated protein 78; GAD65, glutamic acid decarboxylase 65; IGRP, islet-specific glucose-6-phosphatase catalytic subunit-related protein; IAPP, islet amyloid polypeptide; IA-2, islet antigen-2.

Relevant to metabolic pathways, we will summarize the conditions that elicit PTMs, including their role in immunological and biological processes, with specific focus on their implications in T1D. We will particularly highlight the inflammatory PTMs triggered by beta cell stress, the effects of immunometabolic targets/biomarkers on T1D autoimmunity and beta cell metabolism and discuss PTMs-based approaches for preventive and therapeutic options of T1D.

## Beta cell stresses: source and consequence

**Figure 1:**
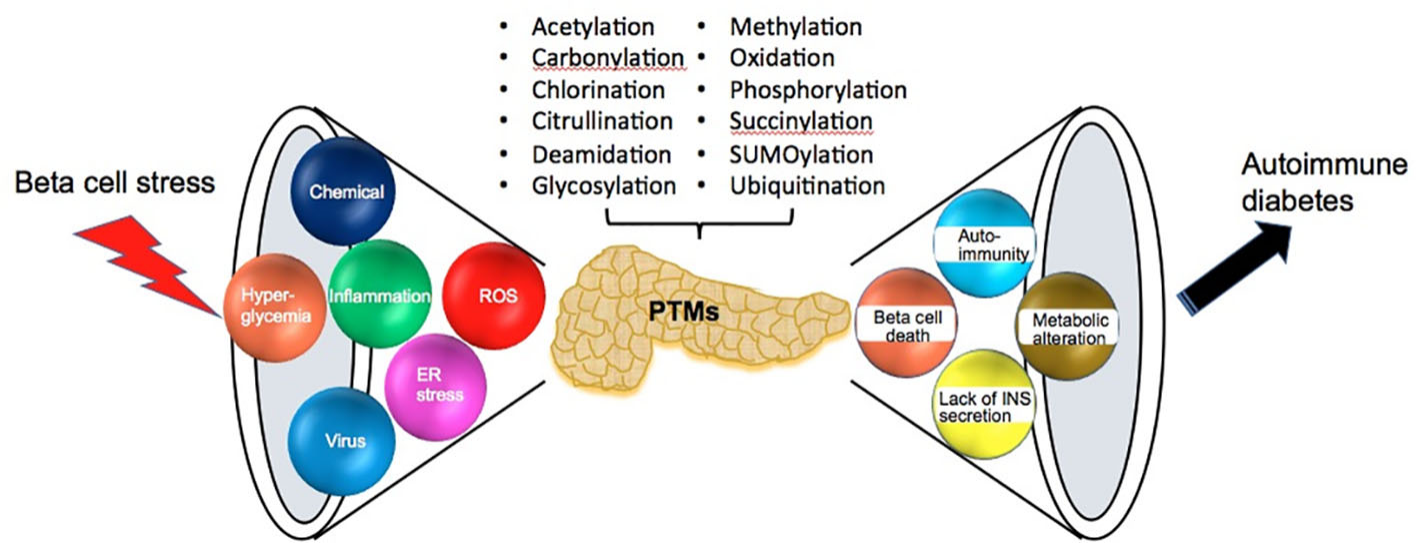
The sources and consequences of beta cell stress. Pancreatic beta cells are confronted with various sources of beta cell stress including viral infection, chemical exposure, hyperglycemia, inflammation, ROS and ER stress. Then beta cell stress results in various posttranslational modifications (PTMs), which cause many effects such as beta cell death, autoimmunity, decreasing insulin secretion and metabolic alteration until the consequence of the onset of autoimmune diabetes.

One pathologic hallmark of juvenile onset T1D is the immense lymphocyte infiltration around and within pancreas islets, i.e., inflammation termed as insulitis. Insulitis includes the massive liberation of proinflammatory cytokines and reactive oxygen species (ROS), a microenvironment that enhances a wide variety of resident protein PTMs. Both experimental and clinical studies demonstrate that beta cell stress-induced PTMs participate in neo-antigen formation, beta cell dysfunction and even beta cell death in the initiation and progression of T1D (**Figure 1**) (11, 56).

Essentially all professional secretory cells rely on endoplasmic reticulum (ER) functions, a protein folding factory, to ensure that accurately folded synthesized proteins find their way to the secretory pathway. However, the process of protein folding is often altered by various cell stresses including viral infection, chemical exposure, heat shock, ROS and inflammation. Improperly folded proteins accumulate in the ER to disrupt ER homeostasis. The ER has finely mechanisms to govern protein quality control by the unfolding protein response (UPR), ER-associated degradation (ERAD) and autophagy (57). One beta cell can synthesize more than 3000 insulin molecules per second (58). Both misfolded proinsulin monomers and aggregates during insulin biosynthesis are primarily cleared by ERAD pathway (59). Therefore, it is not surprising that secretory pathways of beta cell are susceptible to ER stress caused by inflammation and autoimmunity (60–62). For example, ORP150 is an ER resident HSP 70 family chaperone induced by ER stress. Autoantibody against to ORP150 is detected in patients with T1D (63). GRP78 acts as a sentinel to inactivate the UPR pathway by inhibiting ER stress sensor membrane proteins, including protein kinase RNA (PKR)-like ER kinase (PERK), activating transcription factor 6 (ATF6), and inositol-requiring protein 1 (IRE1). While it is not clear that citrullination alters GRP78 activity, it is also found in synovial fluid and antibody against citrullinated GRP78 frequently detected in patients with RA (6, 64). Similarly, citrullinated GRP78 was found in human islets under cytokine-induced stress *in vitro* and antibody against citrullinated GRP78 was also detected in patients with T1D (37). In support of PTMs that drive autoreactive inflammatory processes, there is higher frequency of circulating CD4^+^ T cells against citrullinated GRP78 peptides in T1D PBMC compared to healthy subjects. However, it is clear that the peripheral T cell compartment may not accurately reflect tissue resident T cell populations.

Deletion of the IRE1-X-box–binding protein 1 (XBP1) pathway in pancreatic beta cells results in decreased oxidative folding of proinsulin and insulin secretion along with decreased expression of protein disulfide isomerases (PDIs) (65, 66). Over 30% of proteins require PDI as a chaperone to catalyze disulfide bond formation and facilitate protein folding including preproinsulin, proinsulin and insulin (67). PDIA1, also called prolyl-4-hydroxylase beta (P4Hb), is highly expressed in pancreatic islets and are required for proinsulin oxidative folding *in vitro* (34, 68, 69). Endoplasmic reticulum oxidase 1 (ERO1), another abundant protein expressed in the pancreatic islet, is responsible for recycling reduced PDIA1/P4Hb by FAD cofactor for transferring electrons to oxygen. ERO1-β mutant mice develop impaired glucose-stimulated insulin secretion and decreased insulin content in islets (70). Deficiency of ERO1-β increased cell apoptosis in MIN6 beta cells treated with tunicamycin, an inhibitor of n-glycosylation, resulting in protein misfolding and ER stress (71).

Calcium is essential for the activity of many ER-resident chaperones to ensure accurate protein folding. Sarco/endoplasmic-reticulum calcium ATPase (SERCA) is responsible to pump calcium from cytosol into the ER lumen to maintain higher ER intraluminal Ca^2+^ levels (100-800 µM compared to 100 nM Ca^2+^ in the cytosol) (72). Carbonylation, another PTM amplified in inflamed tissues, leads to a loss of sarco(endo)plasmic reticulum Ca^2+^-ATPase (SERCA2a) activity and diastolic dysfunction in the streptozotocin (STZ)-induced T1D murine model (73). Frequently, ER stress results in Ca^2+^ leakage from ER lumen and then activates Ca^2+^ -dependent PTM enzymes such as tissue transglutaminase 2 (Tgase2) and PADs. Marré et al. reported that chemically-mediated ER stress induced immunogenicity of murine CD4^+^ diabetogenic BDC2.5 T cells mediated by increased Tgase2 activity (74). Recently, Donnelly et al. found that Tgase modified-GAD65 and -IA2 increased the binding affinity of these PTM ligands to their corresponding serum autoantibodies from patients with T1D (75).

Cytosolic PAD enzyme catalyzes the irreversible deimination to convert arginine into citrulline within proteins, a pathway that is closely regulated by calcium. Under the physiologic Ca^2+^ concentration, PADs maintain normal basal activity. When cytosolic calcium concentration increased to 100-fold higher (approximately 1-100µM) above normal physiological concentration in response to cell stresses such as inflammation and ER stress, PAD enzymes become fully activated (76, 77). Among five PAD isozymes, PAD2 has the highest mRNA and protein expression level and activity in the pancreatic islets from C57Bl/6, non-obese diabetes resistance (NOR) and NOD mice (38). However, there is no PAD2 mRNA expression in C57Bl/6, NOR and NOD liver, another major organ for maintaining glucose homeostasis outside of the pancreas (78). Of note, PAD2 and PAD4 are the only PAD isozymes expressed in immune cells and their corresponding enzyme activity in synovial fluid positively correlates with RA tissue inflammation and disease activity (79). Moreover, a pan-PAD inhibitor, BB-Cl-amidine was found to prevent diabetes in the NOD murine model (80). Recently, we have carefully reviewed the role of PAD enzymes in the pathogenesis of T1D development (9).

PADs require reducing conditions for efficient catalytic activity. For example, PAD enzyme in the synovial fluid from patients with RA catalyzes citrullination of human fibrinogen *in vitro* in the presence of reducing agents, dithiothreitol (DTT) or reduced glutathione (GSH) (81). Of note, GSH is the most abundant endogenous antioxidant. In addition, the level of ROS also regulates PAD enzyme activity. Damgaard et al. reported that H_2_O_2_ inhibited the catalytic activity of recombinant human PAD2 and PAD4 *in vitro* (82). Recently, Kim et al. reported that H_2_O_2_ promoted cellular senescence mediated by the inhibition of PAD2 expression in osteoblasts (83). However, how PAD enzyme is regulated and how aberrant PADs activity leads to pathogenic conditions are still not clear. For example, several studies demonstrated that patients with RA have higher oxidative stress and lower GSH level compared to healthy subjects (84–86). Recently, Nagar et al. reported that thioredoxin, the other major redox regulator besides GSH, can activate the enzyme activity of PADs (87). Their study provides one of the mechanisms why citrullination level is increased in patients with RA while the level of GSH, the known co-activator of PADs, is decreased. Collectively, these studies indicate that PAD enzyme activity and citrullination levels in individual tissues and tissue proteins are susceptible to oxidative stress and redox imbalance.

Of note, oxidative stress induced by hyperglycemia and insulitis plays a key role in the onset of T1D and diabetes-related complications of disease. Elevated biomarkers of oxidative stress are frequently detected in tissue, urine and blood from patients with metabolic disorders including T1D and T2D (88–90). It has been hypothesized that beta cells express lower levels of antioxidant enzymes compared to other tissues. The expression of catalase, GPx and both cytosolic Cu/Zn SOD and mitochondrial Mn SOD in mouse islets are lower compared to liver, kidney, brain, heart, lung, skeletal muscle, heart muscle, adrenal gland, and pituitary gland (91, 92). In addition, the expression of catalase and GPx is reduced in human pancreatic beta cells compared to alpha cells. Moreover, beta cell viability is reduced after oxidative stress in H_2_O_2_ or NO treated human islets (93). Consistent with these observations, serum levels of GPx and SOD are reduced in patients with T1D compared to healthy subjects (94). Thus, antioxidants may possess anti-diabetic potential in NOD murine model (95, 96) supporting this therapeutic strategy in patients with T1D (97, 98).

Insulin and its precursors, preproinsulin and proinsulin, also undergo PTM including oxidation and deamidation (39–41). Notably, insulin A-chain (A1-13) with a vicinal disulfide bond between A6-A7 was required for T cell recognition by using a CD4^+^ T cell clone isolated from an HLA DR4^+^ child with autoantibody against insulin (39). Several common PTMs, including citrullination, deamidation, chlorination and oxidation, increase HLA-A*02:01- binding affinity to insulin-B-derived epitopes *in vitro* (45). The deamidation of glutamine catalyzed by Tgase2 is also found to modulate T cell recognition to beta cell autoantigens. Van Lummel et al. reported that deamidation increases epitope binding affinity to HLA DQ by using Tgase2-modified peptides including phogrin, IA-2, IGRP, GAD65 and proinsulin (41). Moreover, there are autoreactive CD4^+^ T cells against deamidated insulin B30-C13 found in patients with early onset T1D.

Protein carbonylation, the major PTM product of oxidative stress, contributes to insulin resistance and metabolic dysfunction in adipose tissue of both animal models and human T1D (99). Hyperglycemia induces oxidative stress, the major stress to trigger and amplify carbonyl modification, and then leads to pancreatic beta cell and endothelial cell dysfunction (100). In adipose tissue, oxidative stress induced GLT4 carbonylation and resulted in GLT4 activity loss (101). Other studies have clearly profiled carbonylated plasma proteins as potential biomarkers in T2D (102–104). Of note, Telci et al. reported that the level of plasma carbonyl PTMs were increased in adolescent and young adult T1D patients compared to the healthy subjects (105). Carbonylated pancreatic amylase and chymotrypsinogen were identified as biomarkers for autoimmune pancreatitis and fulminant T1D, respectively (106, 107). Our recent study also defined a group of pancreatic beta cell proteins with carbonylation, all bound by autoantibodies from human and NOD mice T1D antisera including PDIA1/P4Hb, PDIA2, 14-3-3 protein isoforms, GRP78 and chymotrypsinogen B (35). Of interest, carbonylated PDIA1/P4Hb was found to be an early autoantigen, triggering both autoantibodies and autoreactive T cells in human T1D.

## Glucose metabolic PTM targets: autoreactive T cells and beta cells

T1D is a T cell mediated autoimmune disease that both CD4+ and CD8+ T cells are involved in the selective attack of insulin producing beta cells. Emerging data demonstrate that T cell responses are finely linked to bio-energetic metabolism, glycolysis and oxidative phosphorylation (OXPHOS). In a quiescent state, T cells favor the use mitochondrial OXPHOS to make ATP for basal energy production. Upon antigen stimulation, T cells are reprogrammed to use glycolysis to adjust the increased energy demand to support cell activation, proliferation and differentiation. Inhibition of glycolysis to manipulate T cell autoimmunity has been tested in several autoimmune diseases including SLE, MS, RA and T1D (20, 108, 109). Some of these pathways will be further defined below.

**Figure 2:**
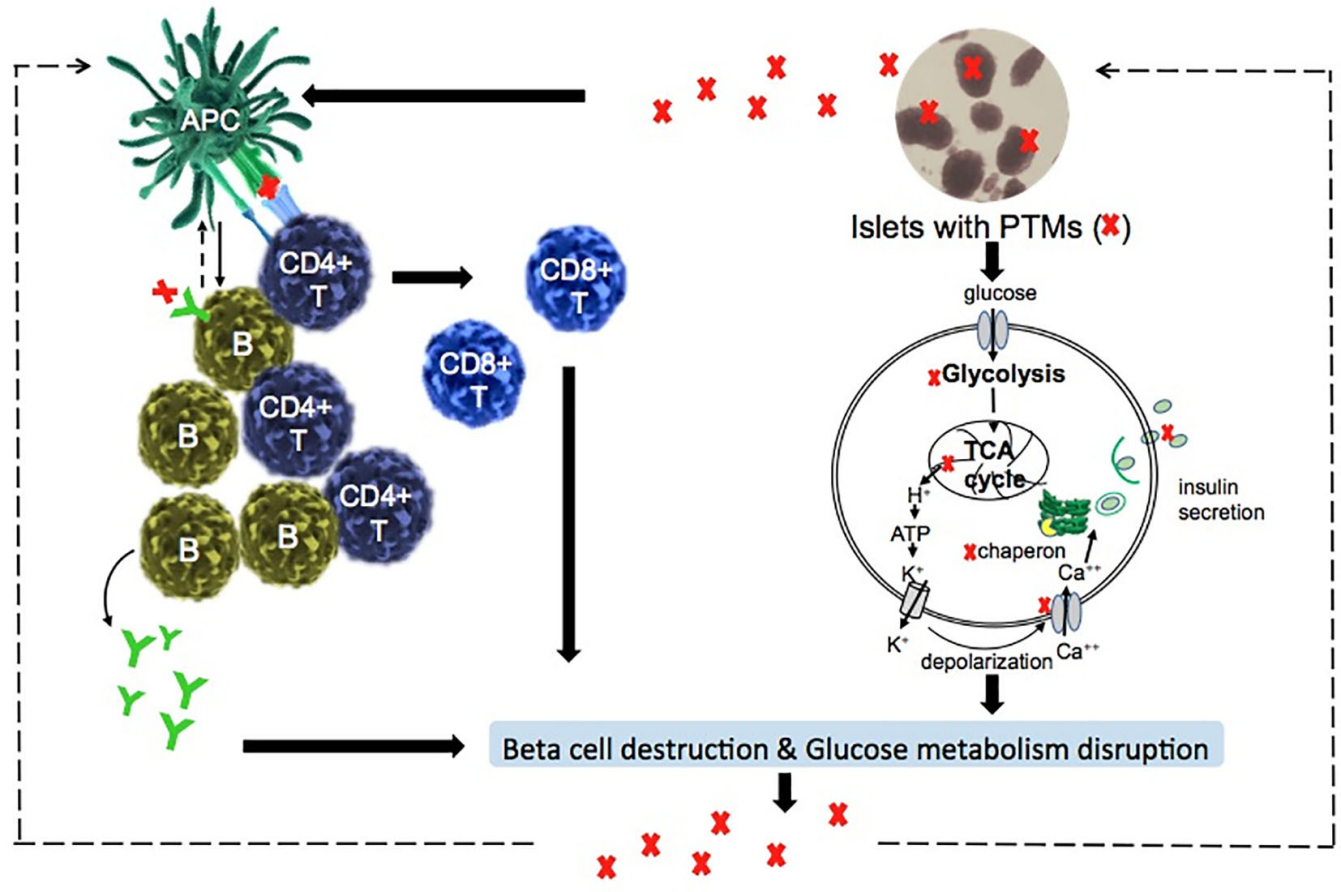
Suggested model of PTMs in the induction of autoreactive responses and dysfunction of beta cells in T1D. T1D is an autoimmune-mediated metabolic disorder. PTMs-associated islet autoantigens are presented by antigen presenting cells (APC), such as dendritic cells and B cells, to activate CD4^+^ T cells and B cells. Activated CD4^+^ T cells interact with B cells and CD8^+^ T cells drive the beta cell destruction. Therefore, glucose homeostasis is disrupted due to the diminishment of insulin-secreting beta cells. On the other hand, PTMs itself directly regulate glucose metabolism such as the biological functions of glucose metabolic enzymes.

The major biological function of pancreatic beta cells is to secret insulin in response to the change of glucose concentration to maintain glucose homeostasis. In the development of T1D, inflammation and oxidative stress amplifies various PTMs within islet self-proteins which then break immune tolerance in addition to altering beta cell metabolism (**Figure 2**) (**Table 1**).

### Glucose transporter

Glucose transporter 1 (GLUT1) facilitates the metabolic switch to glycolysis in activated T cells. 2-Deoxy-D-glucose (2DG), a glucose analog, is taken up by GLUT and then converted to 2DG-6-phosphate by hexokinase in cytoplasm where it is no longer metabolized. 2-DG-6-phosphate accumulating in the cells inhibits hexokinase and phosphoglucose isomerase to then block glycolysis. In comparison to quiescent T cells, activated T cells are more susceptible to 2-DG due to the upregulated GLUT1 expression and glycolytic metabolism. Treatment of NOD mice with 2DG results in the reduction of diabetogenic CD8+ T cells specific to IGRP (NRP-V7 epitope), less lymphocyte infiltration within the islets and improves beta cell granularity (16). GLUT1 blockade therapeutic strategy is also considered for T1D patients that undergo islet transplantation to potentially protect beta cell loss due to graft rejection (110). Glucose uptake is mediated by GLUT2 in rodent beta cells (111). However, it remains controversial if GLUT 1, 2 or 3 are individually critical for glucose uptake in human beta cells (112).

Relevant to glucose uptake, SGLT2 (sodium-glucose co-transporter-2) facilitates renal glucose reabsorption from the circulation. SGLT2 inhibitors reduce renal glucose uptake threshold and have been utilized in patients with T2D to lower plasma glucose levels, with limited risk of hypoglycemia and to prevent cardiovascular complications (17). Since both elevated urine glucose and ROS levels increase SGLT2 activity, several preclinical and clinical studies are ongoing to evaluate the antioxidant effects of SGLT2 inhibitors mainly for patients with T2D but also in streptozotocin (STZ)-induced diabetic murine models (113). Recently, Shyr et al. reported that SGLT2 inhibitors protect from glucotoxicity-induced beta cell failure through mitigation of oxidative and ER stress (114). Of note, several studies evaluated the efficacy and safety of SGLT2 inhibitors in patients with T1D (112, 115).

### Glucokinase

Glucokinase, mainly expressed in the liver and pancreatic beta cells, is the first rate-limiting step of glycolysis in glucose metabolism. However, the metabolic roles of glucokinase in liver and pancreas are fundamentally different for glycogen synthesis and insulin secretion, respectively. Glucokinase (hexokinase IV) belongs to the family of hexokinases. Unlike other hexokinase I-III, glucokinase activity is not regulated by feedback inhibition by its product, glucose-6-phosphate. Glucokinase has ~35-fold lower affinity for glucose (S_0.5_ 7-9 mM) compared to other hexokinases (S_0.5_ ~0.2 mM). In addition, the small fluctuations of its enzyme activity alter the threshold of glucose-stimulated insulin secretion in pancreatic β-cells. Therefore, glucokinase is believed to act as an important glucose sensor by controlling the rate of glucose input into in pancreatic beta cell metabolism.

More than 600 mutations of human glucokinase gene have been identified in patients with glucokinase linked hyperinsulinemic hypoglycemia (PHHI-GK), glucokinase-linked permanent neonatal diabetes (PDNM-GK) and glucokinase-linked maturity-onset diabetes of the young (MODY-GK, also called MODY-2). Several studies also demonstrate that glucokinase activity is regulated by PTMs. For example, polyubiquitination of human glucokinase, both pancreatic isoform 1 and hepatic isoform 2, allosteric activates glucokinase catalytic activity up to 1.4 fold (18). SUMOylation (small ubiquitin-like modifiers) of glucokinase was found in MIN6 and INS-1 murine cell lines and results in increased pancreatic glucokinase stability and activity (19). The STZ-induced diabetic mouse model of T1D exhibits decreased glucokinase expression with hyperglycemia (116). Recently, we found that citrullination decreases the catalytic activity and substrate binding affinity of human pancreatic glucokinase and diminishes glucose stimulated insulin secretion (GSIS) in INS-1E murine cells (10). In addition, citrullinated glucokinase is present in NOD pancreas prior to insulitis and in human islet beta cells exposed to inflammatory cytokines. Moreover, immune self-tolerance is broken by citrullination as indicated by the presence of autoantibodies and autoreactive CD4 T cells against to citrullinated glucokinase in patients with T1D.

Glucose sensing and proliferative capacity differs significantly between immature and mature beta cells, though both secrete insulin. Immature beta cells sense lower glucose concentrations *via* hexokinase 1 and gradually lose proliferative functions as they mature (117). A major difference of metabolic machinery is in the switch of expression from high glucose affinity hexokinase 1 in immature beta cells to low glucose affinity glucokinase (also known as hexokinase 4) in mature beta cells (118). We recently demonstrated that citrullination increases the Km of glucokinase by 2-fold (10). This seemingly small change in Km is nonetheless significant since glucokinase functions in a narrow range of glucose concentration near the Km. While PTMs may trigger increased turnover of modified proteins, there is no known degradation pathway yet identified for protein citrullination. Thus, even small molar changes in irreversible glucokinase citrullination may reflect long term abnormalities in glucose sensing and insulin secretion in individual islets. As defined throughout this review, PTMs that arise may separately alter metabolic processes and/or trigger specific autoimmune responses, and the two outcomes may separately alter or contribute to pathology in the pancreas.

### 6-phosphofructo-2-kinase/fructose-2,6- biphosphatase 3

Besides GLUT1, inhibition of the glycolysis pathway enzymes to modulate T cell autoimmune responses may be an attractive therapeutic strategy for T1D. For example, a small molecule PFK15, a competitive inhibitor of 6-phosphofructo-2-kinase/fructose-2, 6- biphosphatase 3 (PFKFB3), is found to inhibit glycolysis and T cell response to beta cell antigens in diabetogenic CD4+ T cells from NOD.BDC2.5.TCR.Tg mice (20). In addition, PFK15 treatment delayed diabetic onset in the adoptive transfer model of T1D by BDC2.5 CD4+ T cells. Interestingly, PFKFB3 expression is upregulated in beta cells from patients with pre-T1D and T1D compared to non-diabetic subjects (21). Given that several PTMs regulate the biological activity, proteosomal degradation and stability of PFKFB3 in cancer cells (119), it may yet be an important target for therapeutic manipulation of diabetogenic T cells.

### Enolase

Enolase is the glycolytic enzyme that converts 2-phosphoglycerate (2PG) to phosphoenolpyruvate (PEP). The tissue distribution of α-enolase in various autoimmune syndromes has not yet been fully investigated with a potential role in immune mediated tissue pathology. For example, the expression of neuron-specific enolase (NSE) was not detected in the pancreas of autopsied T1D patients, but was present in the islets of non-diabetic subjects (22). Interestingly, anti-α-enolase autoantibodies have been identified in numerous autoimmune diseases such as autoimmune retinopathy, SLE, RA, MS, IBD (120–122). Although PTM modifications of α-enolase with have not yet been reported in autoimmune diabetes, the presence of citrullinated α-enolase in the RA joint and autoantibodies against to citrullinated α-enolase in RA serum suggests that citrullinated α-enolase can initiate and drive chronic inflammatory responses in autoimmune diseases (123).

### Pyruvate kinase

Pyruvate kinase is the last enzyme in glycolysis pathway and produces net ATP and pyruvate. The catalytic activity of PK is tightly regulated by PTMs including phosphorylation, acetylation, citrullination, methylation, succinylation and glycosylation (24–26) as well as through allosteric interaction with F16BP mentioned above. For example, PK activity is increased 2 to 3-fold after *in vitro* citrullination by PAD (124). Our recent studies demonstrate that one pan-PAD inhibitor, YW3-56, can restore cytokine-mediated suppression of insulin secretion upon pyruvate stimulation in INS-1E beta cells (10). The expression of PKM1, PKM2 and α-enolase is down regulated in renal glomeruli from patients with T1D compared to healthy control subjects (23). Pharmacologic activation of pyruvate kinase M2 protects against diabetic nephropathy by increasing glucose metabolic flux and inducing mitochondria biogenesis (27). In rodent and human islets, PK activation accelerates the frequency of metabolic oscillations and increases GSIS *in vivo* (13, 125). Chronic treatment with PK activator also protects islet function on a high fat diet. Upon TCR engagement, PKM2 will translocate into the nucleus of T cells. Treatment with TEPP-46, an allosteric activator of PKM2, blocks its nuclear translocation, inhibits Th1 and Th17 polarization mediated by glycolysis blockade *in vitro* and ameliorates the development of EAE murine model (126). This is another pathway in beta cell metabolism where PTM modification may alter PK biology, yet not affect autoimmune specific responses. There are no defined autoimmune responses to PK in T1D. Thus, PTMs may also singularly affect metabolic components without subsequent autoimmune specific responses (as is the case with PK). The alternative observation that PTMs trigger only autoimmune responses without affecting specific metabolic pathways has been defined in many studies for other self proteins.

### Pyruvate dehydrogenase

Pyruvate is the final product of glycolysis. Once pyruvate enters into mitochondria, it can be metabolized either by pyruvate dehydrogenase (PDH) or pyruvate carboxylase (PC) to enter into TCA cycle metabolism. PDH allows pyruvate to enter into the oxidative TCA pathway through the generation of acetyl CoA in the mitochondrial matrix. PDH activity is regulated by PTMs such as phosphorylation, acetylation and succinylation (28, 29). Beta cell-specific PDH deficient (β-PDHKO) mouse strains develop increased blood glucose level and decreased plasma insulin level in the first month after birth presumably by decreasing glucose oxidation (30). In addition, GSIS was reduced in isolated islets from β-PDHKO mice compared to age-matched control mice. Of note, Zurgil et al. reported that anti-PDH autoantibodies were found in several autoimmune diseases including primary biliary cirrhosis, Sjogren’s syndrome, scleroderma, SLE and RA (127). Interestingly, double knock out the inhibitory PDH kinases in beta cells (that increase PDH activity) actually leads to decreased insulin secretion (125). Thus, the right balance of glucose oxidation versus anaplerosis is required for functional beta cell metabolism (125).

### Pyruvate carboxylase

Comparison of pyruvate oxidation through PDH versus pyruvate carboxylation *via* PC demonstrates that flux through the latter strongly correlates with GSIS in INS1 cells, rodent and human islets (125, 128). While PC is most strongly regulated *via* allosteric activation by acetyl CoA levels (in addition to the direct measures of its flux noted above), its relevance to GSIS has been assessed by knockdown and overexpression approaches. GSIS was not reduced in pyruvate carboxylase siRNA treated INS-1 cells presumably because of incomplete knockdown, while it was suppressed when treated with the chemical inhibitor of PC, phenylacetate (129). In contrast, overexpression of PC in INS-1 cells increases insulin secretion and cell proliferation (31). PC expression is down regulated in islets from patients with T2D compared to non-diabetic subjects (130). To protect human beta cells from inflammation and nitrosative stress, pyruvate carboxylase (PC) is needed for promoting glutathione (GSH) synthesis and suppressing NO synthesis to limit ROS and NO level, respectively (32, 33).

### Protein disulfide isomerase A1/prolyl-4-hydroxylase beta

P4Hb, a member of the PDI family and the beta subunit of a tetramer of prolyl-4-hydroxylase (P4H), is the most abundant ER oxidoreductase for the retention and accurate folding of proinsulin/insulin in pancreatic beta cells (131, 132). Ablation of the thioredoxin activity by chemical modification of PDIA1/P4Hb prevents the refolding of denatured and reduced proinsulin *in vitro* (34). Therefore, PDIA1/P4Hb plays the critical role of proinsulin processing, insulin secretion and protection from ER stress in islet beta cells. Recently, we found that carbonylated P4Hb is elevated in human islets under inflammatory and oxidative stress and is coincident with decreased glucose-stimulated insulin secretion and altered proinsulin to insulin ratios (35). We identified that carbonylated PDIA1/P4Hb serves as an antigenic islet protein supported with the presence of autoantibody and autoreactive T cells against carbonylated PDIA1/P4Hb in patients with T1D. In a small population of early onset T1D patients (n=21, under 1 year of disease duration), we found that 76% patients had either anti-PDIA1/P4Hb alone (11 out of 21 patients) or anti-PDIA1/P4Hb linked with anti-insulin (auto)antibodies (5 out of 21 patients). In contrast, no patient had anti-insulin IgG (auto)antibodies without the presence of anti-PDIA1/P4Hb antibodies, indicating a potential link between these two autoantibody subsets in T1D. Moreover, PDIA1/P4Hb plasma level were increased in pre-diabetic NOD mice and in children with T1D, newly diagnosed within 48 hours (36). In a small cohort of T1D patients (<14 yrs of age) followed longitudinally, some autoantibody responses to P4Hb appear transient, suggesting that antibodies may reflect acute stress in the pancreas. It suggests PDIA1/P4Hb as a potential immunometabolic biomarker for early diagnosis of T1D and also provides mechanistic insight of carbonylated P4Hb into insulin metabolism and neo-epitope formation in the progression of T1D. It is hypothesized that carbonyl modification of P4Hb may cause altered folding of insulin, causing both and accumulation of proinsulin and/or creating an immunogenic misfolded form of insulin itself.

## PTM-based therapeutic approaches

**Figure 3:**
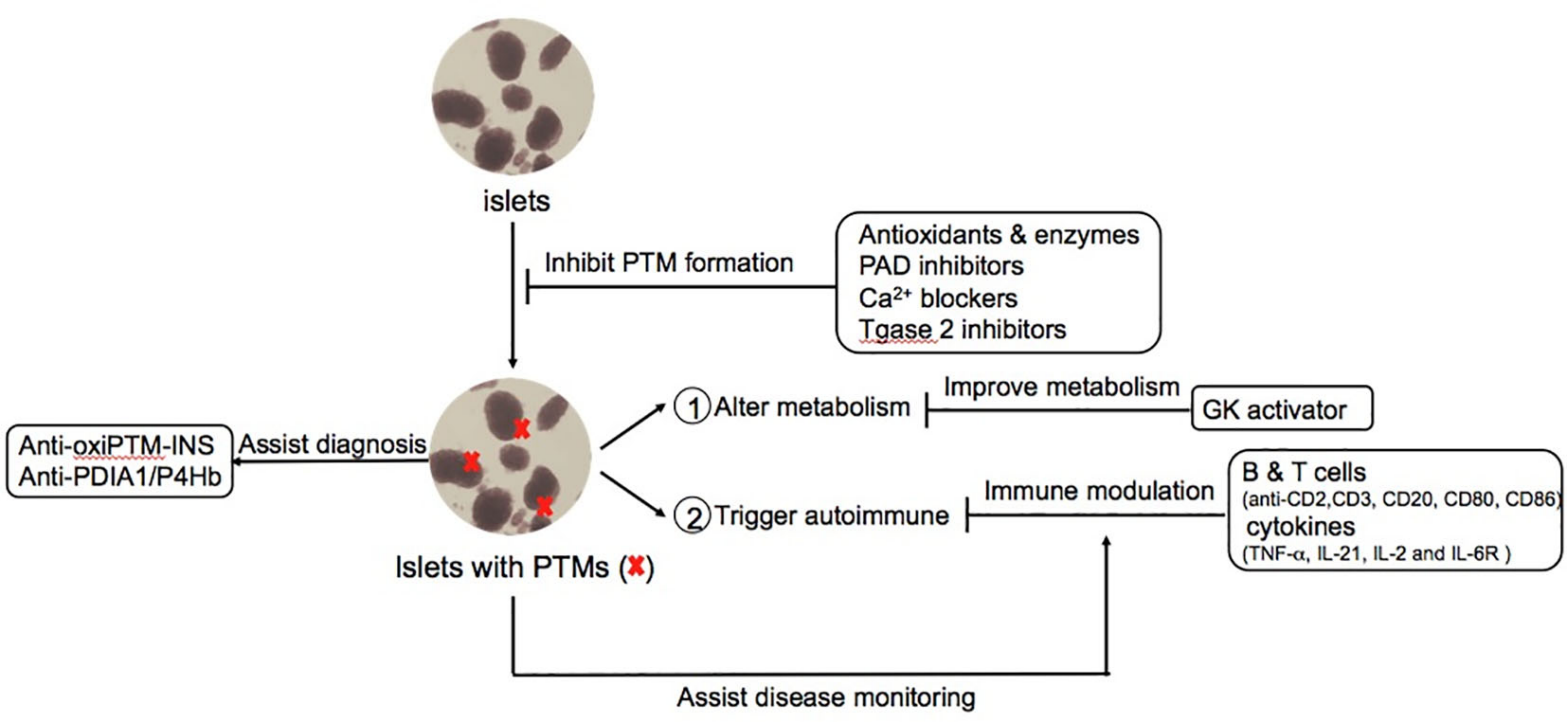
Potential PTMs-based diagnosis and therapeutic approaches for T1D. PTMs provide novel opportunities to mitigate the pathogenesis process of T1D in multiple stages such as avoiding the formation of PTM-associated islet autoantigens, modulating the autoimmune responses and improving glucose metabolism and complications. Moreover, PTM-biomarkers provide better diagnosis and monitoring of disease activity such as autoantibodies against PTMs related proteins.

While exogenous insulin therapy is still the central intervention to treat T1D, a more complete understanding of T1D as a heterogeneous disease with multiple affected immunologic and metabolic pathways encourages versatile modalities to treat, delay and even prevent T1D. Such strategies include immune modulation, islet specific strategies to prevent inflammation, and improved glycemic management. The successful clinical trial of Teplizumab, a FcR non-binding anti-CD3 mAb, reported a delay in the median time to diagnosis of 2 years compared to placebo group for relatives of patients with T1D (133, 134). Importantly, a new era of therapeutic strategy exists with T cell mediated therapy, specifically upon FDA approval of Teplizumab for at-risk T1D individuals to delay the onset of this debilitating disease. Besides anti-CD3 mAb therapy, many attempts of monoclonal antibody and antibody derivatives target on other ligands of T cells and B cells such as CD2, CD20, CD80, CD86 and cytokines such as TNF-α, IL-21, IL-2 and IL-6R are actively ongoing for T1D immune-focused therapy. Other strategies to reduce beta cell stress, maintain islet antigen immune tolerance, sustain glucose homeostasis and even combination therapy are also leading to more therapeutic options for T1D. There are several in-depth reviews recently summarized current and the future therapies for T1D (135–138). Herein, we highlight knowledge gap about potential PTM-based T1D therapy and PTM biomarkers that may reflect diagnosis, disease activity and/or assistance for establishing optimal timing of T1D treatment (**Figure 3**).

Strollo et al. reported that autoantibody against oxidative modified insulin (oxPTM-insulin) and insulin autoantibody (IAA) co-existed in 50% of patients with T1D (42). Of note, 34% of IAA negative T1D patients were oxPTM-insulin positive. In this study, the oxidative modification of insulin antigen includes chlorination of Tyr16 and Tyr26, oxidation of His5, Cys7 and Phe24, and glycation of Lys29 and Phe1 in chain B. Interestingly, some PTMs (citrullination, chlorination, deamidation, and oxidation) can increase the binding affinity of insulin-B-derived peptides on HLA-A*02:01 compared to their counterpart native peptides (45). Strollo et al. also tested the sensitivity and specificity in comparison of oxPTM-insulin antibody with other established T1D autoantibodies (43). Moreover, anti-oxPTM-insulin was observed to precede the onset of T1D in prediabetic children (44). Their studies suggest antibody against oxidative modified insulin as a potential biomarker for better diagnosis compared to current diagnostic T1D autoantibodies and even as a biomarker for prediction of T1D in children. The observations imply that PTM-insulin (oxidation) breaks immune tolerance, leading to autoreactive B and T lymphocytes. Thereafter, subsequent epitope spreading may result in the production of antibody against the non-PTM self insulin protein. As with autoantibody responses to P4Hb, as above, anti-oxPTM-insulin antibodies may be associated with the onset and/or susceptibility of diabetes-associated complications.

The concept of epitope spreading upon breaking of immune tolerance by PTM self proteins has been supported in models of lupus autoimmunity (139–141). The Mamula laboratory demonstrated that elevated isoaspartyl PTM content is found in lupus-prone mice. Particularly, isoaspartyl PTM is associated to lupus T cell proliferative defect in MRL mice (142). Both snRNP D and histone H2B are known lupus autoantigens. Specifically, T cell immune tolerance is broken in isoaspartyl snRNP D immunized mice and subsequent to activate B cells producing antibodies against to both isoaspartyl PTM and naïve forms of snRNP D peptide (140). Moreover, autoantibodies that bind both isoaspartyl and aspartyl form of histone H2B are present in human SLE and lupus-prone MRL/lpr mice, yet another example of epitope spreading (141). It must be emphasized that the clear roles of most PTMs in human disease are not fully understood, notably, whether PTMs are a cause of pathology or a consequence on existing tissue pathology. Moreover, the bias of ex vivo technology in defining T cell specificity from peripheral cells may not accurately reflect tissue resident T cell populations and/or their role in pathology.

In a similar manner as described above, protein carbonylation is the major product of oxidative stress. Recently, we reported that antibodies against carbonyl-PDIA1/P4Hb and native PDIA1/P4Hb often co-exist in patients with established T1D (35). Interestingly, antibody against PDIA1/P4Hb precedes the onset of hyperglycemia in murine NOD mice, as early as 4 weeks of age. In line with the promising anti-CD3 preventive T1D trial in children, PTM relevant autoantibody analysis (either anti-oxPTM-insulin and anti-PDIA1/P4Hb antibodies) may help establish the optimal time for therapeutic immune modulation of high-risk T1D individuals.

Sultan et al. reported that high glucose and oxidative stress increased protein carbonylation and glutathione peroxidase (GPx) activity in HUVECs cells (143). The enhanced GPx activity is due to the compensation of decreased GPx1 protein expression in high glucose or methylglyoxal-treated HUVECs cells. The data suggest that Lys114 carbonylation of GPx1 may alter the substrate H_2_O_2_ biding affinity that increases catalytic activity. It is conceivable that antioxidant approaches to mitigate beta cell stress and/or reduce oxidative PTMs is a potential therapeutic strategy for T1D. Verapamil, a calcium channel blocker, rescued mice from STZ-induced hyperglycemia mediated by preventing beta cell loss (144). Moreover, verapamil decreased thioredoxin-interacting protein (TXNIP) expression in islets from STZ injected mice and TXNIP is one of the important redox regulators in cells. In a phase II clinical trial of 32 adults with recent onset T1D (diagnosed within 3 months), the verapamil treated group had improved glycemic control and mixed-meal-stimulated C-peptide secretion compared to placebo group (145). As noted earlier, calcium is essential for PAD activity. Verapamil fully inhibited Ca^2+^ influx in both A549 and THP-1 cells and fully blocked protein citrullination in A549 cells (146). The effect of verapamil in beta cells on modifying PAD, citrullination, ER stress and other cellular events remains to be more thoroughly investigated for evaluating the therapeutic potential of verapamil in T1D.

Akin to PAD enzymatic activity, Tgase2 (also known as tissue transglutaminase) is a calcium-dependent enzyme that catalyzes deamidation reaction. Anti-Tgase2 antibody serves as a serological marker of celiac disease, thought to be mechanistically associated with T1D (147–149). In particular, Maglio et al. reported anti-Tgase2 antibody deposition in the small intestine of a majority of children with T1D (150). In addition, anti-Tgase2 with combination of IAA, anti-GAD65 and anti-IA2 is found to facilitate screening for pre-T1D and celiac disease (151). Recently, Tgase2 inhibitor, ZED1227, was reported to attenuate gluten-induced small intestinal damage compared to placebo group in a phase II clinical trial of 41 patients with celiac disease (152). However, the therapeutic potential of Tgase2 inhibitors for patients with T1D has not yet been fully investigated.

Several citrulline blockade approaches have been developed and studied in RA. For example, several potent PAD4 specific reversible inhibitors can disrupt mouse and human NET formation (NETosis), thought to be a major source of autoantigens in RA (153–155). Recently, Sodre et al. reported that BB-Cl-amidine, a pan-PAD inhibitor, prevented diabetes development in the NOD murine model mediated by reduced pancreas citrullination level and autoantibody against citrullinated GRP78 (80). Recently, we demonstrated that a PAD2/PAD4 inhibitor, YW3-56, partially restored cytokine-mediated suppression of insulin secretion upon glucose or pyruvate stimulation in INS-1E cells and citrullination disrupted pancreas glucokinase activity (10). While inhibitors of PTMs may be a promising preventative therapeutic approach for T1D, the potential side-effects and/or risks of long term use in young children requires more thorough consideration, given that disease pathology may be chronic in development.

Glucokinase activators have been evaluated in patients with T2D including piragliatin, MK-0941, AZD1656 and dorzagliatin (156, 157). Recently, a phase II clinical trial reported that TTP399, a novel hepatoselective glucokinase activator, lowered HbA_1c_ and reduces hypoglycemia without increasing the risk of ketosis compared to placebo group (158). Last year, the FDA granted a Breakthrough Therapy designation for TTP399 as an adjunctive therapy to insulin for T1D patients based on the promising result by the above clinical trial. Relevant to citrullination, Rituximab, a B cell depleting anti-CD20 antibody, was reported to modulate ACPAs level in patients with RA compared to placebo group (159). In phase II clinical trials, Rituximab delayed the decline of c-peptide, i.e. preserved beta cell function, in patients with T1D compared to placebo group but the effect was transient (160, 161).

## Concluding remarks

Herein, we have identified how specific PTMs may arise in T1D and their consequences on both autoimmunity and metabolic pathways, notably glucose sensing and insulin release. While there are examples of PTMs affecting both autoimmune specificity and metabolism, the effects of PTMs may also be clearly distinct and delineated. For example, a number of PTMs have been noted that trigger autoimmune response without obvious or defined alterations in beta cell metabolism. Conversely, specific PTMs may only affect metabolic pathways in the absence of autoimmune specificity. Thus, therapies that may ‘correct’ PTMs as they arise in T1D may have important consequences to various stages and distinct pathways that characterize T1D. Individual PTMs may be subject to therapeutic manipulation (reversal), while others are permanent and irreversible. Some specific PTMs serve as a common platform for coordinating metabolism and countering beta cell stresses arising from environmental factors (such as diet, virus and chemical) or microenvironmental factors (such as cytokines, ER stress and calcium fluctuation). Similarly, PTMs may serve as biomarkers that may predict and better diagnose early steps in T1D will prove important for designing therapies for preserving and rescuing beta cell functions.

## Author contributions

M-LY, RK and MM conceived the concept and co-wrote the manuscript. All authors contributed to the article and approved the submitted version.

## Funding

M-LY and MM were supported by the JDRF (3-SRA-2017-345-S-B, 1-SRA-2020-977-S-B and 1-INO-2022-1116-A-N) and RK by NIH DK127637.

## Acknowledgments

The authors would like to acknowledge helpful discussion of concepts with colleagues, including Drs. Kevan Herold, Tukiet Lam, Lut Overbergh, Eddie James, Carmella Evans-Molina, Steven Clarke, Hubert Tse and Cate Speake.

## Conflict of interest

The authors declare that the research was conducted in the absence of any commercial or financial relationships that could be construed as a potential conflict of interest.

## Publisher’s note

All claims expressed in this article are solely those of the authors and do not necessarily represent those of their affiliated organizations, or those of the publisher, the editors and the reviewers. Any product that may be evaluated in this article, or claim that may be made by its manufacturer, is not guaranteed or endorsed by the publisher.
